# Semen Modulates Cell Proliferation and Differentiation-Related Transcripts in the Pig Peri-Ovulatory Endometrium

**DOI:** 10.3390/biology11040616

**Published:** 2022-04-18

**Authors:** Jaume Gardela, Mateo Ruiz-Conca, Dominic Wright, Manel López-Béjar, Cristina A. Martínez, Heriberto Rodríguez-Martínez, Manuel Álvarez-Rodríguez

**Affiliations:** 1 Department of Biomedical & Clinical Sciences (BKV), BKH/Obstetrics & Gynecology, Faculty of Medicine and Health Sciences, Linköping University, 58185 Linköping, Sweden; jaume.gardela@uab.cat (J.G.); mateo.ruiz@uab.cat (M.R.-C.); cristina.martinez-serrano@liu.se (C.A.M.); manualvro@gmail.com (M.Á.-R.); 2 Department of Animal Health and Anatomy, Veterinary Faculty, Universitat Autònoma de Barcelona, Bellaterra, 08193 Barcelona, Spain; manel.lopez.bejar@uab.cat; 3 Department of Physics, Chemistry and Biology, Faculty of Science and Engineering, Linköping University, 58185 Linköping, Sweden; dominic.wright@liu.se; 4 College of Veterinary Medicine, Western University of Health Sciences, Pomona, CA 91766, USA

**Keywords:** cell proliferation, cell differentiation, inflammation, interleukins, chemokines, fibroblast growth factors, insulin-like growth factors, pig

## Abstract

**Simple Summary:**

Homeostasis of the uterus after mating is crucial for the subsequent reproductive events. The post-mating inflammatory response is restricted to the uterus, but semen also modulates the expression of other genes involved in regulation along the female reproductive tract, including the oviduct. This study aims to determine if several ejaculate fractions of the pig may modulate cell proliferation and differentiation-related transcripts in different sections of the peri-ovulatory sow reproductive tract. Our data demonstrate that most of the mRNA expression changes of the 144 transcripts tested were induced by mating. Additionally, spermatozoa and seminal plasma also triggered differential expression of the transcripts tested. Finally, our data imply that spermatozoa, seminal plasma components, and the act of mating induce differential mechanisms in the peri-ovulatory female reproductive tract, which are essential for tissue repair.

**Abstract:**

Uterine homeostasis is maintained after mating by eliminating pathogens, foreign cells, and proteins by a transient inflammation of the uterus. Such inflammation does not occur in the oviductal sperm reservoir (utero-tubal junction, UTJ), colonized by a population of potentially fertile spermatozoa before the inflammatory changes are triggered. Semen entry (spermatozoa and/or seminal plasma) modifies the expression of regulatory genes, including cell proliferation and differentiation-related transcripts. Considering pigs display a fractionated ejaculation, this study aims to determine whether different ejaculate fractions differentially modulate cell proliferation and differentiation-related transcripts in the sow reproductive tract during the peri-ovulatory stage. Using species-specific microarray analyses, the differential expression of 144 cell proliferation and differentiation-related transcripts was studied in specific segments: cervix (Cvx), distal and proximal uterus (DistUt, ProxUt), UTJ, isthmus (Isth), ampulla (Amp), and infundibulum (Inf) of the peri-ovulatory sow reproductive tract in response to semen and/or seminal plasma cervical deposition. Most mRNA expression changes were induced by mating. In addition, while mating upregulates the fibroblast growth factor 1 (*FGF1*, *p*-value DistUt = 0.0007; ProxUt = 0.0253) transcript in the endometrium, both its receptor, the fibroblast growth factor receptor 1 (*FGFR1, p*-value DistUt = 2.14 e^−06^; ProxUt = 0.0027; UTJ = 0.0458) transcript, and a potentiator of its biological effect, the fibroblast growth factor binding protein 1 (*FGFBP1*), were downregulated in the endometrium (*p*-value DistUt = 0.0068; ProxUt = 0.0011) and the UTJ (*p*-value UTJ = 0.0191). The *FGFBP1* was downregulated in the whole oviduct after seminal depositions *(p*-value Isth = 0.0007; Amp = 0.0007; Inf = 6.87 e^−05^) and, interestingly, *FGFR1* was downregulated in the endometrium in the absence of semen (*p*-value DistUt = 0.0097; ProxUt = 0.0456). In conclusion, the findings suggest that spermatozoa, seminal components, and the act of mating trigger, besides inflammation, differential mechanisms in the peri-ovulatory female reproductive tract, relevant for tissue repair.

## 1. Introduction

Infertility is a complex component of reproductive health [1]. With the limitations of bioethics on human research, animal models have become a crucial tool for developing such studies [2]. The porcine model presents several similarities with humans, including genetics, anatomy, and physiology [3]. Further research is needed to highlight the importance of the subjacent roles of the molecules involved in infertility in both human and animal models, such as the pig, for the study of reproduction [3,4].

Fertility depends upon a correct balance between pro-and anti-inflammatory components, warranting homeostasis [5,6]. Inflammation is a part of relevant steps of reproductive physiology, present during ovulation, mating, sperm tolerance, placentation, or parturition [7]. The role of semen and seminal plasma (SP) triggering temporary inflammation in the female reproductive tract has been demonstrated in most species [8], including human [9], mice [10], bovine [11], equine [12], porcine [13], and lagomorpha [14]. This temporal post-mating inflammation removes potential pathogens and exogenous SP-proteins, as well as the spermatozoa that do not reach the utero-tubal junction [15]. The endometrium shows essential, hormonally driven, plastic changes along the estrous cycle and embryo implantation [16], primarily influenced by estrogen and progesterone [17], but also influenced by conceptus-derived signals [18]. These endometrial changes involve apoptotic and cell proliferation and differentiation processes [19]. However, whether the semen components (spermatozoa and/or SP) influence expression changes of cell proliferation and differentiation transcripts in the peri-ovulatory reproductive tract is still unknown.

The inflammasomes are large multiprotein oligomers known for contributing to the sensing of pathogen- and damaged-associated molecular patterns triggered by a wide range of substances that emerged during infections, tissue damage, or metabolic imbalance [20,21]. For instance, the nucleotide-binding leucine-rich repeat receptor 3 (NLRP3), a sensor molecule for the formation of inflammasomes, has been pointed as critically involved in the induction of the inflammation [22,23,24], and responsible, at least in part, of preeclampsia in humans when upregulated [25]. The NLRP3-inflammasome interacts with well-characterized inflammation and immune reaction pathways, such as insulin-like growth factors (IGFs) and fibroblast growth factors (FGFs). The IGFs are single-chain polypeptides, structurally related to insulin, playing vital roles in different key processes, including the growth and proliferation of embryos, tissue differentiation, cell survival, and proliferation [26]. The biological effects of IGFs are mediated by a family of cell-surface receptors that includes insulin receptors and IGF receptors (IGFRs) [27]. Additionally, the biological actions of IGFs are modulated by the IGF binding proteins (IGFBPs) [27,28]. The IGF system is essential for preimplantation and placenta development [29]. Local production of IGFs has been reported in the uterus and placenta of several species [30,31,32], including the pig [33,34,35]. The FGFs integrate a large family of polypeptides growth factors with a large variety of functions in development, wound repair, angiogenesis, and metabolism [36,37]. They are sequentially named (FGF1-23) requiring heparan sulfate to activate one of four cell-surface FGF receptors [36]. The FGF family is a multifunctional regulator of differentiation, migration, and cell growth processes [36,37].

The overall aim is to study changes in cell proliferation and differentiation-related transcripts in the peri-ovulatory sow reproductive tract in response to natural mating (i), the insemination with either the first portion of the sperm rich fraction of the ejaculate (ii), the cervical deposition of the SP of the first fraction (iii), or the SP of the complete ejaculate (iv). The hypothesis tested is that semen components (spermatozoa and/or SP) act differentially on specific segments of the genital tract, comparing the tubal sperm reservoir, where fertile spermatozoa escape the post-mating inflammatory response, whereas in the endometrium, the inflammatory cleansing requires tissue repair prior to implantation.

## 2. Materials and Methods

### 2.1. Ethics Permit

Animal handling and experiments were carried out according to the European Community Directive 2010/63/EU, 22 September 2010, and Swedish legislation (SJVFS 2017:40). The study was accepted by the Regional Committee for Ethical Approval of Animal Experiments (Linköpings Djurförsöksetiska nämnd, Linköping, Sweden). Permits number 75-12 (10 February 2012), ID1400 (2 February 2018), and Dnr 03416-2020 (26 March 2020).

### 2.2. Animal Handling and Tissue Collection

Weaned sows (parity 1–3, *n* = 20) and matured boars (9–11 months of age, *n* = 5) of Swedish Landrace breed (*Sus scrofa domestica*) were held under temperature and light control in separate pens at the Translational Medicine Centre (TMC/CBR-3) of Linköping University. Animals were fed with commercial feedstuff, and water was provided *ad libitum*. Sows were exposed to snout-to-snout contact with adjacent boars while pro-estrus and estrus behavior signs were checked by experienced staff, applying back pressure to sows two times per day. When sows presented estrus reflex (first day of behavioral estrus), they were randomly assigned to different experimental groups.

Control females were cervically infused with protein-free Beltsville thawing solution (Control group, *n* = 4), while experimental females were cervically infused with pooled sperm rich-fraction of the ejaculate (Semen-AI group, *n* = 4), pools of sperm-free SP of the whole ejaculate (SP-Total group, *n* = 4), sperm-free SP harvested from pooled sperm-peak fractions (SP-AI group, *n* = 4), or mated with a single male per sow, using 4 different males in total (Natural Mating group, *n* = 4) as previously described [38,39]. After 24 h of each treatment, sows were subjected to general anesthesia. The mucosa of the tubular reproductive tract (right side) was exposed and collected at specific segments: cervix (Cvx), distal uterus (DistUt), proximal uterus (ProxUt), utero-tubal junction (UTJ), isthmus (Isth), ampulla (Amp), and infundibulum (Inf). Mucosal samples were directly plunged into liquid nitrogen and stored in cryovials at −80 °C for mRNA expression analyses.

### 2.3. Collection of Semen and Seminal Plasma

Semen and SP were collected following prior methods [38,39]. The gloved-hand approach was used to collect semen once a week. Sperm concentration and motility were assessed in all samples (QualiSperm; AKYmed, Cheseaux-sur-Lausanne, Switzerland). Ejaculates used for the study had more than 70% motility and 75% spermatozoa with normal morphology. After double centrifugation at 1500× *g* for 10 min and checking for the absence of sperm cells, SP was collected from the total ejaculate or the sperm-rich fraction. 

### 2.4. Transcriptome Analysis and Bioinformatics

Total RNA from reproductive samples was extracted following a TRIzol modified protocol [38,39]. RNA concentration, integrity evaluation, cDNA synthesis, and microarray analyses (GeneChip^®^ Porcine Gene 1.0 ST Array, Affymetrix Inc., 3420 Central Expressway, Santa Clara, CA, USA) were performed according to methods previously described [38,39]. Only samples with RNA integrity values larger than 9 were employed for microarray hybridization. The GeneChip^®^Whole Transcript Plus reagent kit (Affymetrix, Santa Clara, CA, USA) was used to synthesize cDNA (250 ng/reaction). An initial incubation of the hybridization cocktail at 99 °C for 5 min was done after a fall to 45 °C before loading the microarray (GeneChip^®^ Porcine Gene 1.0 ST Array, Affymetrix Inc., 3420 Central Expressway, Santa Clara, CA, USA). The cocktail hybridization solution (130 μL) was then put into each microarray and incubated for 16 h at 45 °C under 60 rotations/min. The hybridized microarray chip was unloaded after incubation and washed and stained with the GeneChip^®^ Fluidics Station 450 (Affymetrix, Santa Clara, CA, USA) before being scanned with the Affymetrix GeneChip^®^ Scanner GCS3000 (Affymetrix, Santa Clara, CA, USA).

Data processing was previously described [38,39], by using the Transcriptome Analysis Console (TAC; version 4.0) from Affymetrix. Briefly, the array chip data were processed using robust multi-array average (RMA) normalization, computing average intensity values by background adjustment, quantile normalization among microarrays, and finally, log2 transformation for extracting the expression values of each transcript in the probe set. The normalized mRNA expression data of the 144 selected transcripts were analyzed, for validation, using the open-source R [40]. Differentially mRNA expression was calculated using a linear model and the empirical Bayes’ approach implemented in the package *limma* [41]. Control type-I errors were checked using a Benjamini–Hochberg false discovery rate (*q* < 0.05) and a principal component analysis-based *p*-value correction. Gene Ontology (GO) terms and pathways were analyzed by the PANTHER classification system (protein annotation through evolutionary relationship) [42] based on the Kyoto Encyclopedia of Genes and Genomes database (KEGG) [43]. Graphical illustration of overrepresented GO terms and pathways was produced with the Cytoscape v3.0.0 application ClueGO v2.0.3 [44] and principal component analysis (PCA) and heatmaps were performed by using the ClustVis web tool [45].

## 3. Results

**Figure 1:**
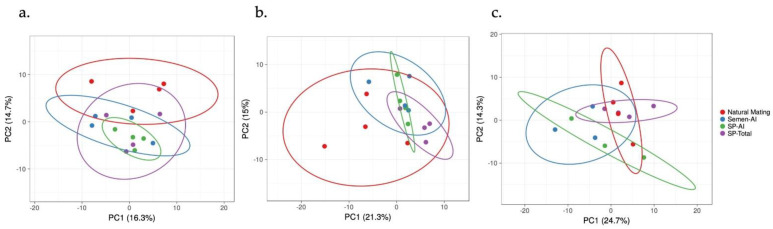
Principal component analysis of the genes included in this study of natural mating, Semen-AI, SP-AI; and SP-Total groups. (**a**). DistUt: distal uterus; (**b**). ProxUt: proximal uterus; (**c**). UTJ: utero-tubal junction.

The PCA analysis of the 144 transcripts depicted an appropriate division of the treatment groups (Figure 1 and Figure S1) and the grouping factor by heatmaps of the chosen genes was conserved across tissues analyzed (Figure S2).

**Figure 2:**
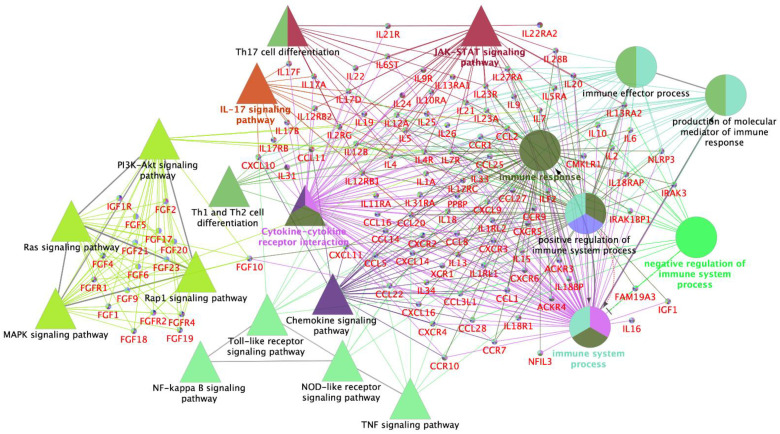
Schematic representation of altered transcripts of interest among all tissues and treatments. The over-representation of functional categories was carried out using the Cytoscape v3.0.0 (ClueGo v2.0.3). Gene Ontology (GO) subgroups biological process are depicted as circles. Terms are grouped based on common genes function (kappa score) and discriminated by colors. The size of the nodes shows the degree of significance (low to high significance, respectively). ClueGo parameters of biological process; GO tree levels, 2–4 (from 0); minimum number of genes, 1; minimum percentage of genes, 1.2; GO term fusion; GO term connection restriction (kappa score), 0.4; GO term grouping, initial group size of 2 and 50% for group merge.

The official names of the genes and the *Sus scrofa* annotation in the KEGG pathways database [43] was used to find the differentially expressed genes with different biological pathways and GO biological process subgroups (Figure 2).

**Figure 3:**
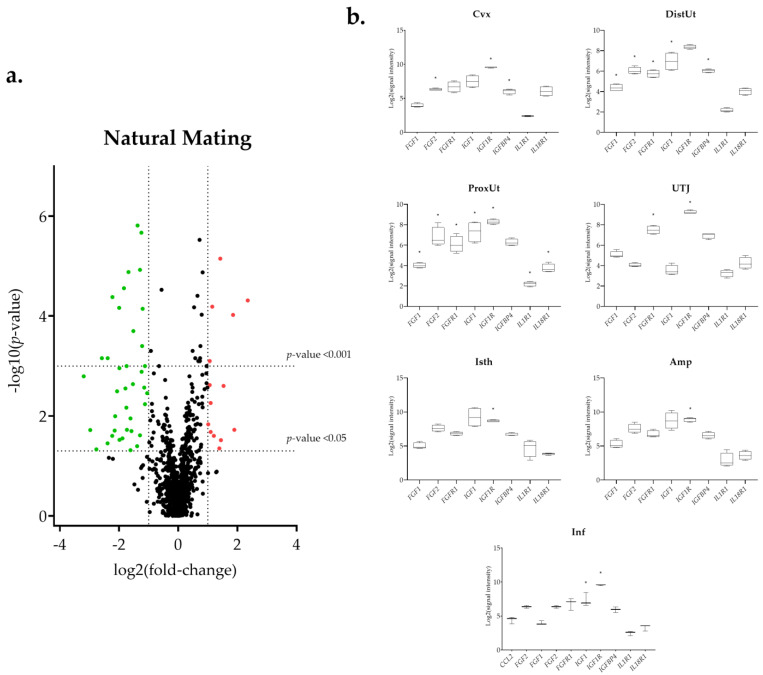
Summary of the transcriptomic analysis in the natural mating group. (**a**) Volcano plot representation of differential expression analysis of transcripts in the natural mating versus negative control comparison. Red dots represent upregulated transcripts and green dots downregulated transcripts. Black dots represent all transcripts that showed an altered expression profile. The x-axis shows the log2 fold-changes in expression and the y-axis the statistical significance (−log10 *p*-value). (**b**) Box-plot representation of the log2 signal intensity of the selected transcripts by tissue: Cvx: cervix; DistUt: distal uterus; ProxUt: proximal uterus; UTJ: utero-tubal junction; Isth: isthmus; Amp: ampulla; and Inf: infundibulum. * *p* < 0.05 relative to negative control.

**Figure 4:**
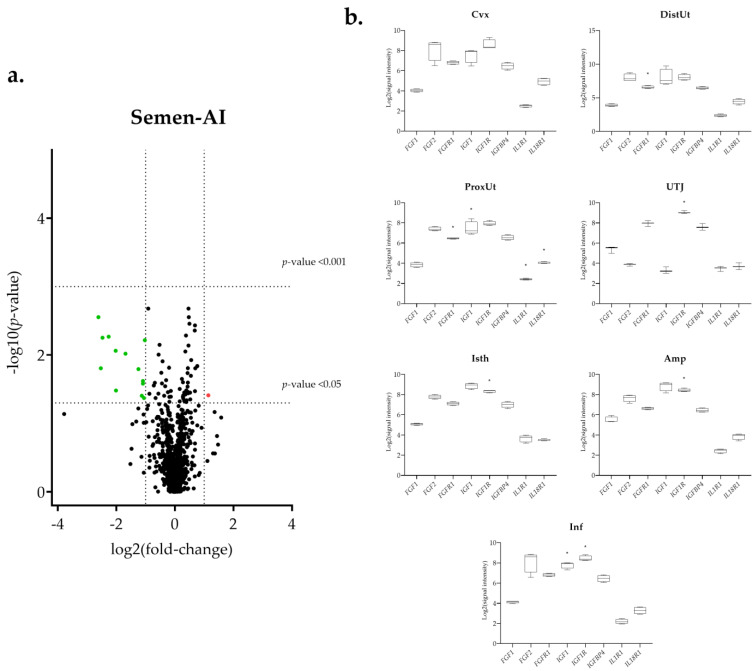
Summary of the transcriptomic analysis in the Semen-AI group. (**a**) Volcano plot representation of differential expression analysis of transcripts in the Semen-AI versus negative control comparison. Red dots represent upregulated transcripts and green dots downregulated transcripts. Black dots represent all transcripts that showed an altered expression profile. The x-axis shows the log2 fold-changes in expression and the y-axis the statistical significance (−log10 *p*-value). (**b**) Box-plot representation of the log2 signal intensity of the selected transcripts by tissue: Cvx: cervix; DistUt: distal uterus; ProxUt: proximal uterus; UTJ: utero-tubal junction; Isth: isthmus; Amp: ampulla; and Inf: infundibulum. * *p* < 0.05 relative to negative control.

**Figure 5:**
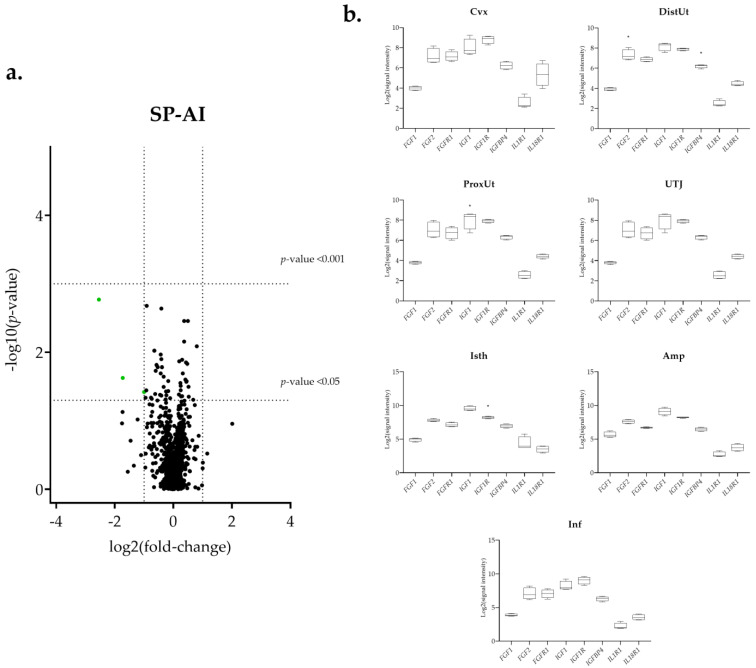
Summary of the transcriptomic analysis in the SP-AI group. (**a**) Volcano plot representation of differential expression analysis of transcripts in the SP-AI versus negative control comparison. Green dots represent downregulated transcripts. Black dots represent all transcripts that showed an altered expression profile. The x-axis shows the log2 fold-changes in expression and the y-axis the statistical significance (−log10 *p*-value). (**b**) Box-plot representation of the log2 signal intensity of the selected transcripts by tissue: Cvx: cervix; DistUt: distal uterus; ProxUt: proximal uterus; UTJ: utero-tubal junction; Isth: isthmus; Amp: ampulla; and Inf: infundibulum. * *p* < 0.05 relative to negative control.

**Figure 6:**
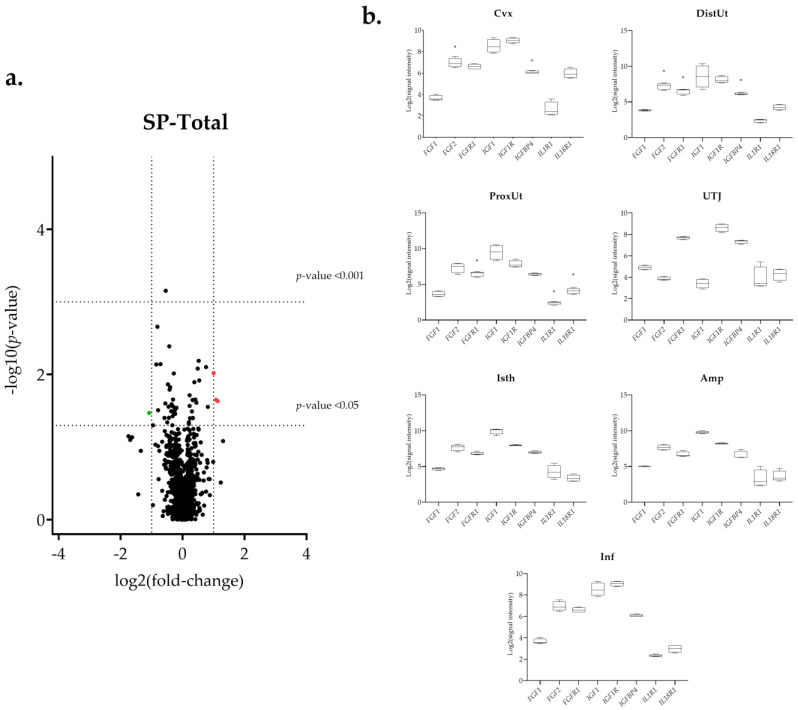
Summary of the transcriptomic analysis in the SP-Total group. (**a**) Volcano plot representation of differential expression analysis of transcripts in the SP-Total versus negative control comparison. Red dots represent upregulated transcripts and green dots downregulated transcripts. Black dots represent all transcripts that showed an altered expression profile. The x-axis shows the log2 fold-changes in expression and the y-axis the statistical significance (−log10 *p*-value). (**b**) Box-plot representation of the log2 signal intensity of the selected transcripts by tissue: Cvx: cervix; DistUt: distal uterus; ProxUt: proximal uterus; UTJ: utero-tubal junction; Isth: isthmus; Amp: ampulla; and Inf: infundibulum. * *p* < 0.05 relative to negative control.

The differential expression of the 144 selected transcripts showed the changes were in response to the natural mating, mostly downregulated (Figure 3a and Table S1). Moreover, some transcriptomic changes were found in the groups containing the first portion of the sperm-rich fraction (Semen-AI; Figure 4a) and only SP, either from the first fraction of the ejaculate (SP-AI; Figure 5a) or from the entire ejaculate (SP-Total; Figure 6a).

### 3.1. The IGF1 Transcript Downregulation Was Strongly Marked in Mating Compared to Semen-AI and SP-AI

The insulin-like growth factor 1 (*IGF1*) presented a common downregulation in the ProxUt for the mating, Semen-AI, and SP-AI groups (Figure 3b, Figure 4b, and Figure 5b). Additionally, it was also downregulated in the DistUt (natural mating group) and Inf (natural mating and Semen-AI groups; Figure 3b and Figure 4b). In contrast, its receptor *IGF1R*, especially in the natural mating group, was upregulated in all tissues but not in the DistUt (Table S1). The insulin-like growth factor receptor 1 (*IGFR1*) transcript was also upregulated in the UTJ, Isth, Amp, and Inf in the Semen-AI group, whereas it was only upregulated in Isth in the SP-AI group (Table S1).

### 3.2. Interleukin Modulation along the Sow Reproductive Tract in Response to Semen and SP

The atypical chemokine receptor 3 (*ACKR3*) transcript expression was downregulated in the mating group (ProxUt, DistUt, and Isth, *q* < 0.05; Cvx and UTJ, *p* < 0.05), the SP-AI group (DistUt, *p* < 0,05), and the SP-Total group (Cvx, *p* < 0.05). The *IL-10* transcript (anti-inflammatory) was upregulated in the UTJ, but only for the natural mating group (Figure 3b). The interleukin 1 alpha (*IL-1A*, pro-inflammatory) was downregulated in the natural mating group (Cvx, DistUt, and ProxUt; Table S1). One of its receptors, *IL1RL1*, was downregulated in the ProxUt for the natural mating, Semen-AI, and SP-Total groups, whereas the *IL1R1* was upregulated, only for the natural mating group, in the UTJ, Isth, and Inf (Table S1). The nuclear factor interleukin 3 regulated (*NFIL3*, activates transcription from the interleukin-3 promoter in T-cells) was upregulated in the UTJ, and the entire oviduct (Isth, Amp, and Inf) in response to natural mating, whereas it was downregulated in the Cvx, ProxUt, and Inf in SP-Total (Table S1). In contrast to the downregulation of *CXCL8* (pro-inflammatory) in the Cvx and ProxUt (natural mating group) and UTJ in Semen-AI, the receptor *IL17RB*, of its main inductor (*IL17E*), was upregulated in the DistUt, UTJ, Isth, Amp, and Inf (natural mating group) and only in the Isth (Semen-AI group) (Table S1). Interleukin 34 (*IL-34*, pro-inflammatory) was upregulated in the natural mating group in the DistUt, ProxUt, Amp, and Inf (Table S1). Interleukin 6 (*IL-6*, showing both pro- and anti-inflammatory actions) was downregulated in the Amp (natural mating and Semen-AI groups; Figure 3b and Figure 4b). *IL-6* receptor alpha-like was downregulated, only for the mating group in the ProxUt, Amp, and Inf (Table S1). Interleukin 19 (*IL-19*, anti-inflammatory) was downregulated in the Cvx and DistUt for the natural mating group (Figure 3b), and in the DistUt (SP-Total group; Figure 6b), whereas it was upregulated in the Isth in both sperm-free groups (SP-AI and SP-Total groups; Figure 5b and Figure 6b).

### 3.3. Sperm-Free Treatments and Mating Triggered a Common Downregulation of FGF2 Transcript in the Distal Uterus

The *FGF2* was downregulated in the DistUt of the mating, SP-AI, and SP-Total groups, and also in the cervix (Cvx) of the natural mating and SP-Total groups (Figure 3b, Figure 5b and Figure 6b). Additionally, the natural mating group also triggered downregulation in the ProxUt (Figure 3b). Its specific receptor, the fibroblast growth factor receptor 1 (*FGFR1*), was downregulated in the DistUt and ProxUt of the natural mating, Semen-AI, and SP-Total groups (Table S1). Moreover, it was also downregulated in the UTJ, but only for the natural mating group.

## 4. Discussion

Semen and SP can trigger significant gene expression changes in the female reproductive tract [8]. In the present study, we have explored the modulation of 144 mRNA transcripts related to cell proliferation and differentiation in the sow reproductive tract in triggered by semen and sperm-free SP. For this purpose, we analyzed the changes in response to the four experimental groups included in the study: natural mating (i), artificial insemination of the sperm rich fraction (ii), infusion of sperm-free SP of the sperm rich fraction (iii), and infusion of sperm-free SP of the entire ejaculate (iv), using commercial microarrays to assess gene expression [46]. Overall, the natural mating group induced most of the transcriptomic changes. However, some key differentially expressed transcripts were shared between experimental groups, suggesting a common underlying physiological mechanism at work across these treatments. It might be related, at least in part, to the signaling agents present in the SP that have been pointed out to promote molecular and cellular changes into the female reproductive tract of several species [8,47]. Our results confirm changes in the expression of cell proliferation and differentiation-related transcripts in the female reproductive tract in response to both sperm and SP. However, independent verification of such selected transcripts should be performed, especially for markers that have a less than 2-fold difference expression.

Several chemokines are produced throughout the estrous cycle and pregnancy, playing essential roles in regulating endometrial functions for estrous cyclicity and establishing and maintaining pregnancy [48,49,50]. The atypical chemokine receptors (ACKRs) are similar to traditional chemokine receptors but regulate chemokine activity by limiting their spatial availability or removing them from in vivo sites [51]. Because of their structural inability to couple to G proteins, ACKRs bind chemokines with high affinity but do not trigger cell migration [52]. Expression regulation of *ACKR1*, *ACKR2*, *ACKR3*, and *ACKR4* transcripts has been demonstrated in the endometrium of pigs during the estrous cycle [53], maternal-fetal interface [54,55], and pregnancy [53]. Our results confirm that this modulation is also present during the male-female interaction along the female reproductive tract, suggesting that the ACKRs may play essential roles in regulating chemokine action in the endometrium and oviduct during the male-female interaction.

*ACKR3* binds to *CXCL12*, the ligand of *CXCR4*, and *CXCL11*, one of the ligands of *CXCR3* [52]. Our results show that the expression of *ACKR3* mRNA was lower in the cervix, endometrium, utero-tubal junction, and isthmus after mating. Additionally, the expression of *ACKR3* mRNA was lower in the distal uterus and cervix after infusion of sperm-free SP of the sperm rich fraction and the infusion of sperm-free SP of the entire ejaculate, respectively. The in vitro inhibition of *ACKR3* reduces M2 macrophages receptor expression on monocytes, suggesting that *ACKR3* may play a role in fine-tuning immunological tolerance at the fetal-maternal interface [56]. Moreover, because *ACKR3* acts as a decoy receptor for *CXCL11* and *CXCL12* [52], the decrease in *ACKR3* mRNA expression after mating is likely to allow *CXCL11* and *CXCL12* to perform their functions of immune cell recruitment during the post-mating inflammatory response. However, *CXCL11* mRNA expression was lower in the cervix, proximal uterus, and ampulla after mating, and no changes in *CXCL12* mRNA expression were reported.

*CXCR4* is the most regularly expressed chemokine receptor and is involved in various physiological and pathological situations involving cell movement and cell proliferation [57]. *ACKR3* modulates the *CXCR4* expression and signaling activity by forming heterodimers with *CXCR4*, producing conformational rearrangements in G-protein complexes and partially to β-arrestin rather than classical G-protein coupled receptor signaling in response to *CXCL12* binding [58]. After mating, we reported lower *ACKR3* mRNA expression along the female reproductive tract, whereas *CXCR4* mRNA expression was higher. This dual modulation of both receptors may regulate an appropriate response after mating. However, the factors regulating *ACKR3* and *CXCR4* in the female reproductive tract during the male-female interaction still need to be determined. Although more research is needed to understand the precise functions of the ACKRs and their ligands during the male-female interaction, our findings suggest that the expression and regulation of these chemokine receptors may be necessary in regulating endometrial and oviductal functions for the post-mating inflammatory response.

The IGFs play crucial roles in tissue differentiation, cell survival, proliferation [26], and myometrial steroidogenic activity [59]. The main focus on pig reproduction research has been on the IGFs expression in the uterus [33,34,35,59,60,61], although some research focused on oviductal IGFs expression [62,63]. Our data showed a consistent downregulation of the *IGF1* expression in the proximal uterus in response to mating, the artificial insemination of the sperm-rich fraction, and the infusion of seminal plasma from the sperm-peak portion. In contrast, upregulation of *IGF1R* transcript in oviductal tissues in the mating and artificial insemination of the sperm-rich fraction was found. Previous research confirmed that *IGF1* contributes to increase the intrauterine content of estrogen during the peri-implantation period, and the relative abundance of *IGF-1R* mRNA transcript and protein is altered in the porcine endometrium depending on the reproductive status of the sow [59]. Our results suggest an implication of the IGF system not only during the peri-implantation period but also in the peri-ovulatory stage. Additionally, these changes are present in the myometrium and the oviduct, and it seems that the sperm-containing treatments trigger the changes observed in the oviduct.

In conjunction with other growth factor systems, the FGF system appears to be involved in a paracrine network to effectively establish and maintain pregnancy in the pig [64]. Two of the most studied members of the FGF family (*FGF1* and *FGF2*), and their receptors (*FGFR1IIIc* and *FGFR2IIIc*) are constitutively expressed in the porcine uterus and oviduct [64,65,66]. Our study detected downregulation of *FGF2* expression in the uterus after mating and sperm-free SP treatments during the peri-ovulatory stage. In the same way, we observed a downregulation of *FGFR1* expression in sperm-containing treatments and sperm-free SP of the whole ejaculate. Otherwise, sperm-containing treatments induced upregulation of *FGFR2* and the isoform *FGFR2IIIc* expression in the uterus and oviduct during the peri-ovulatory stage. In addition, we observed an upregulation of *FGF1* expression in the endometrium induced by mating. FGF receptors play different biological importance for a functional endometrium and are promoted differently by estradiol (*FGFR1IIIc*) and progesterone (*FGFR2IIIc*) [66]. The luteal phase of the estrous cycle also changes the expression of the FGF ligands in the bovine endometrium, and this modulation is linked with the expression of their receptors in the embryo [67]. Additionally, previous research demonstrated that SP upregulates the expression of *FGF2* in the porcine oviduct [68]. Our results suggest that *FGF1*, *FGFR2*, and *FGFR2IIIc* isoform play more essential roles in the pre-/peri-ovulatory stage of the porcine endometrium and endosalpinx compared to *FGFR1* and *FGF2*, which seems required in early stages of pregnancy [65].

*FGF2* has been described as an adipokine that may exacerbate the inflammatory response in adipocytes through *NLRP3* inflammasome activation [69]. *NLRP3* is a critical component of the inflammasome activated by microbial pathogens and endogenous molecules [70]. The activation of *NLRP3* requires extracellular inflammatory stimuli, which induces the transcription of *NLRP3* and controls post-translational modifications that license receptor activation [71]. Once activated, the inflammasome leads to an induction of pro-IL-1β, and activation of caspase-1, promoting the secretion of the pro-inflammatory cytokines *IL-1β* and *IL-18* [72]. The wide variety of stimuli activating *NLRP3* inflammasome [70,71] and the immune regulatory modulation produced in the female reproductive tract after entry of seminal components [13] could be related to our results that showed an upregulation of the *NLRP3* transcript in the distal uterus after mating. However, the expression of the *IL-18* transcript was downregulated in the same tissue fragment, and no modulation of the *IL-1β* transcript was found. Additionally, previous results demonstrated a downregulation of the *caspase-1* transcript in the endometrium of sows after 24 h of mating [73]. In addition, while the *NLRP3* transcript between the mating and the sperm-free SP of the entire ejaculate infusion was upregulated, it was not present in the infusion of sperm-free SP-rich fraction group. Thus, our results suggest that the SP components from the entire ejaculate, not only from the rich sperm fraction, are responsible for the *NLRP3* transcript modulation, similarly to the common upregulation of the *NLRP3* transcript that was present in the previously described sperm reservoir of the sow reproductive tract, the utero-tubal junction [74,75].

Even though more experiments are required, is it therefore likely that *NLRP3* inflammasome, including FGFs and IGFs-related transcripts, may be involved in sperm selection, facilitate tissue remodeling, or elimination of damaged endometrial cells after mating. An inflammatory response is induced in the uterus after mating in response to semen; however, this response facilitates the efficient removal of spermatozoa and induces transient immune tolerance to seminal antigens because of components present in the SP [76]. Additionally, it has been suggested that the post-mating inflammation and the immunological events that follow the beginning of inflammation have a role in establishing immune tolerance to embryonic antigens and subsequent embryonic development in domestic animals [76]. Similarly, the inflammasomes are essential in the maternal-fetal interface remodeling of endometrium during the early stage of implantation and are a defense against pathogen infections [77]. However, when overly activated, they can be highly pathological, inducing several reproductive diseases [77]. Overall, our results suggest a hypothetical involvement of cell proliferation and remodeling genes in the physiological interaction between the female reproductive tract and sperm, including the seminal fluid.

## 5. Conclusions

In conclusion, we have demonstrated that the seminal fluid, including the SP alone, triggers differential mRNA transcript expression in proliferation and differentiation-related pathways in the sow reproductive tract. Our data suggested that as early as the periovulatory stage, relevant factors present in sperm and/or SP produce changes in mRNA expression in the female genital tract, including the FGFs and the IGFs (and their specific receptors) as well as some interleukin-immune related genes, such as the interaction between *IL-18* and *NLRP3*, and the *ACKR3* and *CXCR4*. However, further studies are required to precisely characterize the mechanism by which the sperm (and/or SP) affects the transcript expression and whether this knowledge may enhance the fertility performance in assisted reproductive techniques.

## Data Availability

Not applicable.

## Supplementary Materials

The following supporting information can be downloaded at: https://www.mdpi.com/article/10.3390/biology11040616/s1, Table S1: Fold change of the differential mRNA expression (*p* < 0.05, red: False Discovery Rate, *q* < 0.05) in the different segments of the sow reproductive tract (cervix to infundibulum), 24 h post-treatment. Figure S1 Principal Component Analysis of the genes included in this study of natural mating (red); Semen-AI (blue); SP-AI (green); and SP-Total (purple) treatment groups. a. Cvx: cervix; b. Isth: isthmus; c. Amp: ampulla; and d. Inf: infundibulum. Figure S2. Heatmap of the genes included in this study of natural mating (red); Semen-AI (blue); SP-AI (green); and SP-Total (purple) treatment groups. a. Cvx: cervix; b. DistUt: distal uterus; c. ProxUt: proximal uterus; d. UTJ: utero-tubal junction; e. Isth: isthmus; f. Amp: ampulla; and g. Inf: infundibulum.
