# Supplementary material for: Semen Modulates Cell Proliferation and Differentiation-Related Transcripts in the Pig Peri-Ovulatory Endometrium
Source: Biology (Basel). 2022 Apr 18;11(4):616. doi: 10.3390/biology11040616 (PMC9029625; doi:10.3390/biology11040616)
Supplement: Supplementary file 1 [file biology-11-00616-s001.zip › biology-1649199-supplementary.pdf]

biology

*Article*

# Semen Modulates Cell Proliferation and Differentiation-Related Transcripts in the Pig Peri-Ovulatory Endometrium

**Jaume Gardela [1,2,†], Mateo Ruiz-Conca [1,2,†], Dominic Wright [3], Manel López-Béjar [2,4], Cristina A. Martínez [1], Heriberto Rodríguez-Martínez [1,*,‡] and Manuel Álvarez-Rodríguez [1,2,‡]**

1 Department of Biomedical & Clinical Sciences (BKV), BKH/Obstetrics & Gynecology, Faculty of Medicine and Health Sciences, Linköping University, SE-58185 Linköping, Sweden; jaume.gardela@uab.cat (J.G.); mateo.ruiz@uab.cat (M.R.-C.); cristina.martinez-serrano@liu.se (C.A.M.); manualvro@gmail.com (M.Á.-R.)
2 Department of Animal Health and Anatomy, Veterinary Faculty, Universitat Autònoma de Barcelona, 08193 Bellaterra, Spain; manel.lopez.bejar@uab.cat (M.L.-B.)
3 Department of Physics, Chemistry and Biology, Faculty of Science and Engineering, Linköping University, 58185 Linköping, Sweden; dominic.wright@liu.se
4 College of Veterinary Medicine, Western University of Health Sciences, Pomona, CA 91766, USA
* Correspondence: heriberto.rodriguez-martinez@liu.se
† These authors contributed equally to this work.
‡ These authors contributed equally to this work.

**Table S1.** Fold change of the differential mRNA expression ($p < 0.05$, red: False Discovery Rate, $q < 0.05$) in the different segments of the sow reproductive tract (cervix to infundibulum), 24 h post-treatment.

| Gene Symbol | Natural Mating | | Semen-AI | | SP-AI | | SP-TOTAL | |
|---|---|---|---|---|---|---|---|---|
| | **UP** | **DOWN** | **UP** | **DOWN** | **UP** | **DOWN** | **UP** | **DOWN** |
| ***ACKR1*** | - | Isth (-1.78) | Cvx (1.61) | - | - | - | - | - |
| ***ACKR2*** | Amp (1.19), Inf (1.26) | Cvx (-1.44) | - | - | - | - | - | - |
| ***ACKR3*** | - | Cvx (-1.9), DistUt (-3.21), ProxUt (-2.59), UTJ (-2.33), Isth (-2.45) | - | - | - | DistUt (-1.37) | - | Cvx (-1.76) |

| | | | | | | | | |
|---|---|---|---|---|---|---|---|---|
| ***ACKR4*** | UTJ (1.23), Inf (1.57) | - | ProxUt (1.39) | - | - | - | Inf (1.17) | Cvx (-1.29) |
| ***CCL1*** | - | - | - | - | - | - | - | - |
| ***CCL11*** | Isth (1.39) | - | UTJ (1.22), Isth (1.25) | - | - | ProxUt (-1.13) | - | ProxUt (-1.23) |
| ***CCL14*** | DistUt (1.76) | - | Cvx (1.27) | - | - | - | - | - |
| ***CCL16*** | - | - | UTJ (1.21) | Isth (-1.14) | - | - | DistUt (1.15) | - |
| ***CCL19*** | Inf (1.26) | - | - | - | Inf (1.25) | - | Amp (1.18) | - |
| ***CCL2*** | - | Cvx (-5.23) | - | - | - | - | - | - |
| ***CCL20*** | - | Cvx (-6.81) | Inf (1.18) | - | Inf (1.18) | - | - | - |

| | | | | | | | | |
|---|---|---|---|---|---|---|---|---|
| ***CCL21*** | - | - | - | - | - | - | - | - |
| ***CCL22*** | Amp (1.3) | - | - | - | - | - | - | - |
| ***CCL23*** | - | - | Amp (1.36) | - | Amp (1.43) | - | - | - |
| ***CCL25*** | - | - | - | - | - | Inf (-1.08) | - | - |
| ***CCL27*** | ProxUt (1.26) | Isth (-1.2) | - | - | - | - | - | - |
| ***CCL28*** | Cvx (2.3), Inf (3.72) UTJ (2.72), Amp (2.89) | - | - | UTJ (-2.08) | - | - | - | - |
| ***CCL3L1*** | - | - | - | - | - | - | - | - |

| | | | | | | | | |
|---|---|---|---|---|---|---|---|---|
| ***CCL4*** | Inf (1.37) | - | - | - | - | - | - | - |
| ***CCL5*** | Inf (1.13) | - | - | UTJ (-1.48) | - | - | - | - |
| ***CCR1; XCR1*** | - | Cvx (-3.06) | - | - | - | - | - | - |
| ***CCR10*** | ProxUt (1.21), Inf (1.21) | - | - | - | - | - | - | - |
| ***CCR7*** | ProxUt (1.23) | - | - | - | - | - | - | - |
| ***CCR9*** | - | Cvx (-1.3) | - | - | - | - | - | Cvx (-1.25) |
| ***CMKLR1*** | - | - | Cvx (1.61), ProxUt (1.35) | - | - | - | - | - |

| | | | | | | | | |
|---|---|---|---|---|---|---|---|---|
| ***CXCL10*** | - | Cvx (-4.69), Amp (-3.69) | - | Amp (-3.22) | - | Amp (-1.9) | UTJ (2.19) | - |
| ***CXCL11*** | - | Cvx (-3.45), ProxUt (-4.44), Amp (-2.45) | - | Amp (-2.37) | - | - | - | - |
| ***CXCL12*** | - | - | - | - | - | - | - | - |
| ***CXCL14*** | Amp (2.62), Inf (1.77) | - | - | Cvx (-2.19), UTJ (-5.56), Isth (-4.07) | - | UTJ (-3.31), Isth (-5.82) | - | - |
| ***CXCL16*** | - | - | - | UTJ (-2.13) | - | - | - | - |
| ***CXCL2*** | - | Cvx (-3.96) | - | UTJ (-6.12) | - | - | - | - |

| | | | | | | | | |
|---|---|---|---|---|---|---|---|---|
| ***CXCL8; LOC100620730*** | - | Cvx (-4.38), ProxUt (-3.06) | - | UTJ (-5.78) | - | - | - | - |
| ***CXCL9*** | UTJ (1.43) | - | ProxUt (2.21) | - | - | - | UTJ (2) | - |
| ***CXCR1*** | - | - | - | - | - | Cvx (-2) | - | - |
| ***CXCR2*** | - | - | - | - | - | - | - | - |
| ***CXCR3*** | Inf (1.21) | - | Cvx (1.37) | - | - | - | - | - |
| ***CXCR4*** | DistUt (1.68), UTJ (1.75), Isth (5.1), Amp (1.29), | - | - | - | - | - | - | - |

| | | | | | | | | |
|---|---|---|---|---|---|---|---|---|
| | Inf (2.22) | | | | | | | |
| ***CXCR5*** | Amp (1.27), Inf (1.21) | - | - | - | - | - | - | - |
| ***CXCR6*** | - | - | - | - | - | - | - | - |
| ***FAM19A1*** | - | - | Amp (1.31), Inf (1.38) | - | - | ProxUt (-1.26) | - | - |
| ***FAM19A3*** | - | UTJ (-1.33) | - | - | Isth (1.23) | - | Isth (1.15) | UTJ (-1.46), Amp (-1.36) |
| ***FAM19A4*** | - | Cvx (-3.35), DistUt (-1-97), ProxUt (-2.36), | - | Cvx (-2.13), ProxUt (-2.04) | - | ProxUt (-1.67) | Inf (1.3) | - |

| | | | | | | | | |
|---|---|---|---|---|---|---|---|---|
| | | UTJ (-2.07), Isth (-1.57), Amp (-2.18), Inf (-2.3) | | | | | | |
| ***FGF1*** | DistUt (1.67), ProxUt (1.23) | - | - | - | - | - | - | - |
| ***FGF10*** | DistUt (1.63), Isth (1.62) | - | - | - | - | - | - | - |
| ***FGF12*** | - | Isth (-1.77) | - | - | - | - | - | - |
| ***FGF14*** | Cvx (1.47), ProxUt (1.18), Isth (2.14) | - | - | - | - | - | - | - |

| | | | | | | | | |
|---|---|---|---|---|---|---|---|---|
| ***FGF17*** | ProxUt (1.17) | DistUt (-1.12) | - | DistUt (-1.1) | - | Isth (-1.35) | - | - |
| ***FGF18*** | Inf (1.55) | - | - | - | - | - | - | - |
| ***FGF19*** | - | Amp (-1.16) | - | - | - | - | - | - |
| ***FGF2*** | - | Cvx (-2.89), DistUt (-3.57), ProxUt (-2.62) | - | - | - | DistUt (-1.54) | - | Cvx (-1.94), DistUt (-1.24) |
| ***FGF20*** | - | DistUt (-1.29) | - | - | - | - | - | - |
| ***FGF21*** | ProxUt (1.32) | - | - | - | - | - | - | - |
| ***FGF23*** | - | - | - | - | - | - | - | - |
| ***FGF4*** | - | - | - | - | - | - | - | - |

| | | | | | | | | |
|---|---|---|---|---|---|---|---|---|
| ***FGF5*** | - | - | ProxUt (1.12) | - | - | - | - | - |
| ***FGF6*** | - | - | - | - | - | - | - | - |
| ***FGF9*** | - | DistUt (-1.43) | - | DistUt (-1.44) | - | DistUt (-1.33), Isth (-1.33) | - | Isth (-1.35) |
| ***FGFBP1*** | - | Cvx (-9.15), DistUt (-3.37), ProxUt (-3.97), UTJ (-7.86), Isth (-5.99), Amp (-5.22), Inf (-3.99) | - | - | - | - | Amp (1.76) | - |

| | | | | | | | | |
|---|---|---|---|---|---|---|---|---|
| ***FGFR1*** | - | DistUt (-2.37), ProxUt (-2.22), UTJ (-1.28) | - | DistUt (-1.38), ProxUt (-1.58) | - | - | - | DistUt (-1.22), ProxUt (-1.41) |
| ***FGFR1OP*** | - | Amp (-1.19), Inf (-1.33) | - | - | - | Isth (-1.18) | Cvx (1.23) | - |
| ***FGFR1OP2*** | - | ProxUt (-1.29) | - | - | - | - | - | - |
| ***FGFR2*** | Cvx (2.02), UTJ (1.28), Isth (1.19) | - | ProxUt (1.46) | - | - | - | - | - |
| ***FGFR2IIIC*** | UTJ (1.57), | - | UTJ (1.7) | - | - | - | - | - |

| | | | | | | | | |
|---|---|---|---|---|---|---|---|---|
| | Isth (1.85) | | | | | | | |
| ***FGFR4*** | UTJ (1.28), Amp (1.2) | - | - | - | - | - | - | - |
| ***IGF1*** | - | DistUt (-2.99), ProxUt (-4.19), Inf (-4.67) | - | ProxUt (-4.8), Inf (-1.88) | - | ProxUt (-1.86) | - | - |
| ***IGF1R; LOC100623509; LOC100622436*** | Cvx (1.93), ProxUt (1.56), UTJ (1.49), Isth (1.65), Amp (1.77), | - | UTJ (1.36), Isth (1.3), Amp (1.12), Inf (1.41) | - | Isth (1.2) | - | - | - |

| | | | | | | | | |
|---|---|---|---|---|---|---|---|---|
| | Inf (1.73) | | | | | | | |
| ***IGF2BP2*** | - | - | Inf (1.44) | UTJ (-1.65) | - | - | - | - |
| ***IGF2R; LOC100739014*** | Inf (1.15) | - | - | - | - | - | - | - |
| ***IGFALS*** | - | - | - | - | - | - | - | - |
| ***IGFBP1*** | - | - | - | - | - | - | - | - |
| ***IGFBP4*** | - | Cvx (-1.4), DistUt (-1.36) | - | - | - | DistUt (-1.13) | - | Cvx (-1.47), DistUt (-1.32) |
| ***IGFBP7*** | - | DistUt (-1.15), ProxUt (-1.39) | - | - | - | - | - | Cvx (-1.64) |
| ***IL10*** | UTJ (1.45) | - | - | - | - | - | - | - |

| | | | | | | | | |
|---|---|---|---|---|---|---|---|---|
| ***IL10RA; LOC100622546*** | Amp (1.43), Inf (1.29) | - | Cvx (1.43), UTJ (1.28) | - | - | - | - | - |
| ***IL11RA*** | DistUt (1.2), ProxUt (1.27), | - | - | - | - | - | - | - |
| ***IL12A*** | - | DistUt (-1.22) | - | - | - | - | - | - |
| ***IL12RB1*** | Amp (1.23), Inf (1.1) | - | ProxUt (1.25) | - | - | - | - | - |
| ***IL12RB2*** | - | - | - | - | - | - | - | - |
| ***IL13*** | - | - | - | - | - | - | - | Cvx (-1.37) |
| ***IL13RA1*** | Amp (1.34), | PorxUt (-1.28) | - | - | - | - | - | - |

| | | | | | | | | |
|---|---|---|---|---|---|---|---|---|
| | Inf (1.4) | | | | | | | |
| ***IL13RA2*** | - | - | - | - | Inf (1.37) | - | - | - |
| ***IL15*** | - | - | Cvx (1.35),<br>Inf (1.47) | - | Inf (1.23) | - | Cvx (1.4),<br>DistUt (1.33) | - |
| ***IL-15L*** | Amp (1.45) | - | Amp (1.22) | - | Amp (1.19) | - | - | - |
| ***IL16*** | Inf (1.3) | - | - | - | - | DistUt (-1.2) | - | - |
| ***IL17A*** | - | - | - | - | - | - | Cvx (1.17) | - |
| ***IL17B*** | - | - | UTJ (1.31) | - | - | - | - | - |
| ***IL17D*** | ProxUt (1.33),<br>UTJ (1.35), | - | - | - | - | - | - | - |

| | | | | | | | | |
|---|---|---|---|---|---|---|---|---|
| | Isth (1.94), Amp (1.33) | | | | | | | |
| ***IL17RB*** | DistUt (1.19), UTJ (2.14), Isth (2.68), Amp (2.09), Inf (1.6) | - | Isth (1.6) | ProxUt (-1.26) | - | - | - | - |
| ***IL17RC*** | - | - | - | - | Cvx (1.6) | - | - | Inf (-1.24) |
| ***IL17RD*** | - | UTJ (-1.36), Amp (-1.18), Inf (-1.61) | - | - | - | - | - | - |

| | | | | | | | | |
|---|---|---|---|---|---|---|---|---|
| ***IL17RE; LOC106505669*** | Cvx (1.47), DistUt (1.4), ProxUt (1.27) | - | ProxUt (1.38) | - | Cvx (1.39), ProxUt (1.19) | - | Cvx (1.45) | - |
| ***IL17REL*** | ProxUt (1.26) | - | - | - | - | - | - | - |
| ***IL18*** | - | DistUt (-1.8), ProxUt (-1.85) | - | - | - | Proxut (-1.49) | - | - |
| ***IL18BP*** | - | - | - | - | - | - | - | Cvx (-1.26) |
| ***IL18R1*** | - | ProxUt (-2.18) | - | ProxUt (-1.61) | - | - | - | ProxUt (-1.5) |
| ***IL18RAP*** | Isth (1.22) | Cvx (-1.92) | - | - | - | Cvx (-1.57), Distut (-1.52) | - | - |

| | | | | | | | | |
|---|---|---|---|---|---|---|---|---|
| ***IL19*** | - | Cvx (-1.29),<br>DistUt (-1.36) | - | - | Isth (1.35) | - | Isth (1.36) | DistUt (-1.19) |
| ***IL1A*** | - | Cvx (-3.33),<br>DistUt (-1.87),<br>ProxUt (-1.58) | - | - | - | - | - | - |
| ***IL1B1; IL1B2*** | Inf (1.54) | - | - | - | - | - | - | - |
| ***IL1B2*** | - | Cvx (-1.33) | - | - | - | Isth (-1.35) | - | Amp (-1.73) |
| ***IL1RAPL1*** | - | - | - | - | UTJ (1.36) | - | - | - |
| ***IL1RAPL2*** | DistUt (1.28) | - | Inf (1.21) | - | - | - | - | - |
| ***IL1RL1*** | - | ProxUt (-1.34) | - | ProxUt (-1.18) | - | - | - | ProxUt (-1.15) |

| | | | | | | | | |
|---|---|---|---|---|---|---|---|---|
| ***IL1RL2*** | - | - | - | - | - | - | - | - |
| ***IL2*** | - | - | UTJ (1.32) | - | - | - | - | - |
| ***IL20*** | - | - | - | ProxUt (-1.15) | - | - | - | - |
| ***IL21*** | - | - | - | Isth (-1.14) | UTJ (1.29) | - | - | Isth (-1.18) |
| ***IL21R*** | - | - | - | - | - | - | - | - |
| ***IL24; LOC100621682*** | - | Inf (-1.45) | - | DistUt (-1.1) | - | - | - | - |
| ***IL25*** | - | - | - | - | - | - | - | - |
| ***IL26; LOC100620108*** | - | - | - | - | UTJ (1.14) | - | - | - |
| ***IL27RA*** | - | - | Cvx (1.62) | - | - | - | - | - |
| ***IL28B*** | ProxUt (1.22) | - | - | - | - | - | - | - |

| | | | | | | | | |
|---|---|---|---|---|---|---|---|---|
| ***IL2RG*** | Isth (1.42) | - | - | - | - | - | - | - |
| ***IL31*** | Amp (1.31) | - | - | - | UTJ (1.33) | - | - | - |
| ***IL31RA*** | DisUt (1.25), ProxUt (1.37), UTJ (1.76), Inf (1.23) | - | - | - | - | - | - | - |
| ***IL33*** | - | ProxUt (-1.84) | - | - | Inf (1.74) | - | UTJ (2.11), Amp (1.69) | - |
| ***IL34*** | DistUt (1.19), ProxUt (1.22), Amp (1.26), | - | - | - | - | - | - | - |

| | | | | | | | | |
|---|---|---|---|---|---|---|---|---|
| | Inf (1.68) | | | | | | | |
| ***IL4*** | - | - | - | - | - | - | - | - |
| ***IL4I1*** | - | - | - | - | - | UTJ (-1.33) | - | - |
| ***IL4R*** | Amp (1.1), Inf (1.08) | - | - | - | - | DistUt (-1.31) | - | - |
| ***IL5*** | - | Amp (-1.23) | - | Amp (-1.23) | - | - | - | Amp (-1.19) |
| ***IL5RA*** | Amp (1.29) | - | - | - | - | - | - | - |
| ***IL-6; IL6*** | - | Amp (-1.24) | - | Amp (-1.36) | - | - | - | - |
| ***IL6ST*** | Isth (1.27) | Cvx (-1.31) | - | - | - | - | - | - |
| ***IL7; LOC100622266*** | - | ProxUt (-1.31) | DistUt (1.28) | - | - | - | - | - |

| | | | | | | | | |
|---|---|---|---|---|---|---|---|---|
| ***IL7R*** | - | ProxUt (-1.43) | - | - | - | - | - | - |
| ***IL9*** | - | - | - | - | - | - | Inf (1.43) | - |
| ***IL9R*** | - | - | - | - | - | - | - | - |
| ***ILF2*** | - | Amp (-1.37), Inf (-1.85) | - | Inf (-1.47) | - | - | Isth (1.26) | - |
| ***ILF3*** | - | - | - | - | - | - | - | - |
| ***IRAK1*** | - | - | - | - | - | - | - | - |
| ***IRAK1BP1*** | - | - | DistUt (1.28) | Cvx (-1.83) | - | - | - | - |
| ***IRAK3*** | - | - | - | ProxUt (-1.34) | - | - | - | - |

| | | | | | | | | |
|---|---|---|---|---|---|---|---|---|
| ***IRAK4*** | - | DistUt (-1.19), ProxUt (-1.48) | - | UTJ (-1.29) | Inf (1.29) | - | - | - |
| ***LOC100516362; LOC106509761 (IL1R2-like)*** | Isth (1.21) | - | - | - | Isth (1.15) | - | - | - |
| ***LOC100518910 (IL17RA-like)*** | - | - | - | - | - | DistUt(-1.26), Isth (-1.45) | - | DistUt (-1.28) |
| ***LOC100622457 (IL20R$\alpha$)*** | - | Isth (-2.86) | - | UTJ (-4.04) | - | Isth (-1.7) | - | Amp (-2.11) |
| ***LOC100626904 (IL1R1)*** | UTJ (1.7), Isth (1.94), | - | - | - | - | - | - | - |

| | | | | | | | | |
|---|---|---|---|---|---|---|---|---|
| | Inf (1.5) | | | | | | | |
| ***LOC100628187; IL22*** | - | - | - | - | - | - | - | - |
| ***LOC102162067 (IL6Rα-like)*** | ProxUt (1.32), Amp (1.38), Inf (1.41) | - | - | - | - | - | - | - |
| ***NFIL3*** | UTJ (1.71), Isth (3.61), Amp (2.09), Inf (1.98) | - | - | - | - | - | - | Cvx (-1.8), ProxUt (-1.34), Inf (-1.39) |

| | | | | | | | | |
|---|---|---|---|---|---|---|---|---|
| ***NLRP3*** | DistUt (1.34), UTJ (1.66) | - | - | - | - | - | UTJ (1.25) | - |
| ***PPBP; LOC100524561; LOC100620821*** | - | - | - | - | - | DistUt (-1.19) | - | - |

* Cvx: cervix; DistUt: distal uterus; ProxUt: proximal uterus; UTJ: utero-tubal junction; Isth: isthmus; Amp: ampulla; and Inf: infundibulum. Natural mating: sows mated with a boar; Semen-AI: sows artificially inseminated with the sperm-peak portion extended to 50 mL with Beltsville thawing solution (BTS); SP-AI: sows cervically infused with the sperm-free seminal plasma (SP) from pooled sperm-peak portion (50 mL); SP-TOTAL: sows cervically infused with the sperm-free SP of the whole ejaculate (50 mL). All treatments were compared with controls. $p < 0.05$ relative to negative control.

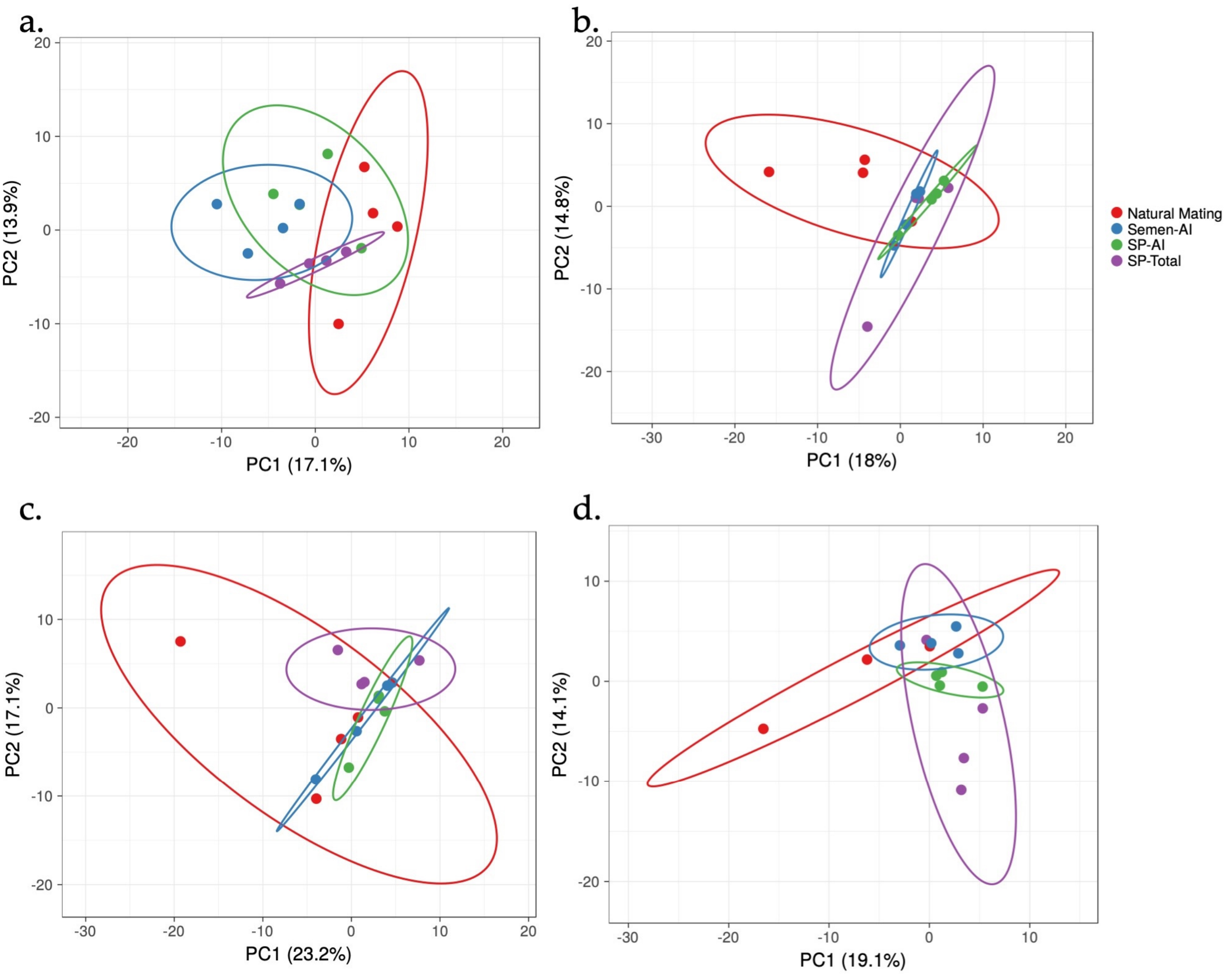


**Figure S1.** Principal Component Analysis of the genes included in this study of natural mating (red); Semen-AI (blue); SP-AI (green); and SP-Total (purple) treatment groups. a. Cvx: cervix; b. Isth: isthmus; c. Amp: ampulla; and d. Inf: infundibulum.

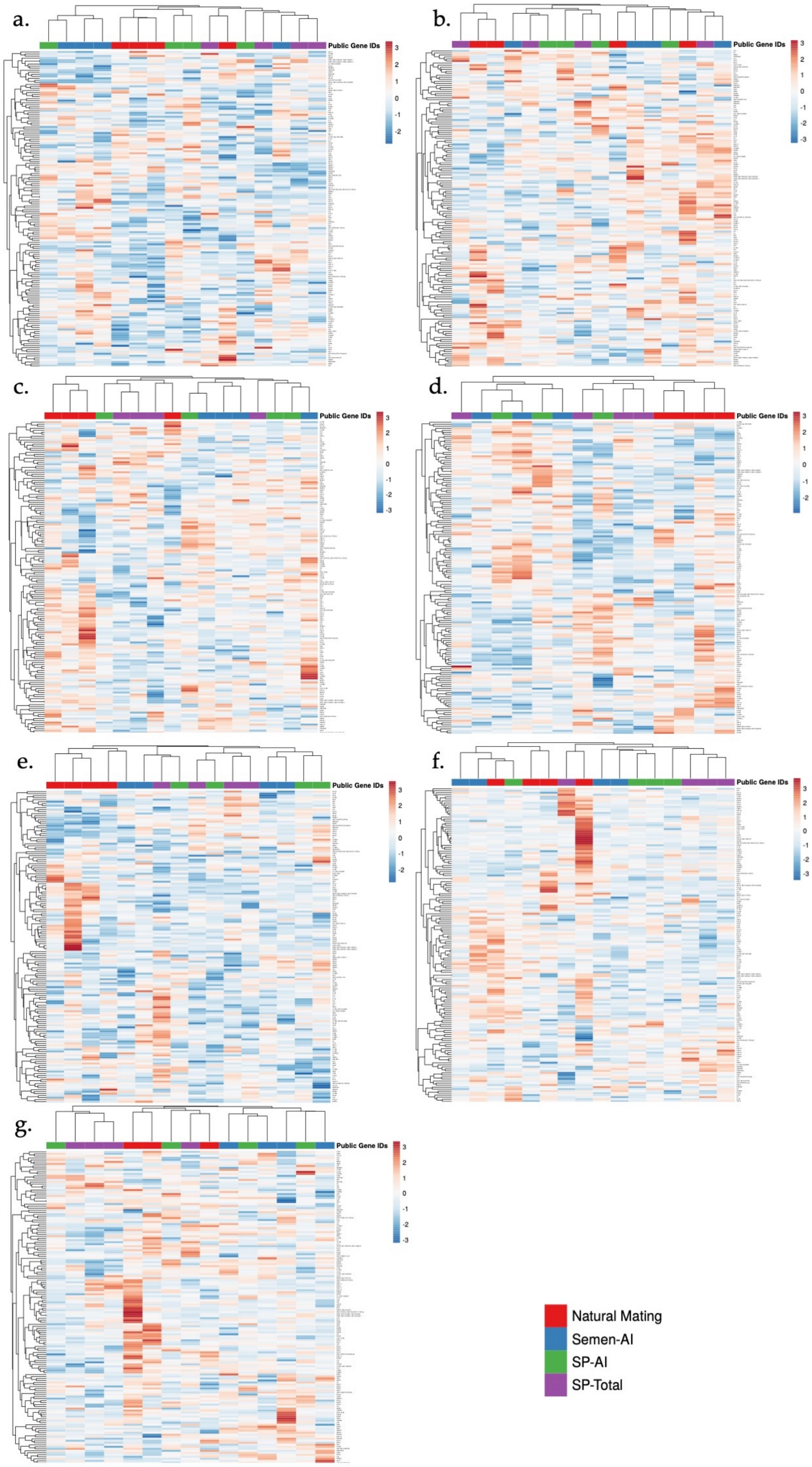


**Figure S2.** Heatmap of the genes included in this study of natural mating (red); Semen-AI (blue); SP-AI (green); and SP-Total (purple) treatment groups. a. Cvx: cervix; b. DistUt: distal uterus; c. ProxUt: proximal uterus; d. UTJ: utero-tubal junction; e. Isth: isthmus; f. Amp: ampulla; and g. Inf: infundibulum.
